# The GerW Protein Is Not Involved in the Germination of Spores of *Bacillus* Species

**DOI:** 10.1371/journal.pone.0119125

**Published:** 2015-03-19

**Authors:** Jose Cruz-Mora, Abigail Pérez-Valdespino, Srishti Gupta, Nilumi Withange, Ritsuko Kuwana, Hiromu Takamatsu, Graham Christie, Peter Setlow

**Affiliations:** 1 Department of Molecular Biology and Biophysics, University of Connecticut Health Center, Farmington, Connecticut, United States of America; 2 Department of Chemical Engineering and Biotechnology, University of Cambridge, Cambridge, United Kingdom; 3 Faculty of Pharmaceutical Sciences, Setsunan University, Hirakata, Osaka, Japan; University of Wisconsin, Food Research Institute, UNITED STATES

## Abstract

Germination of dormant spores of *Bacillus* species is initiated when nutrient germinants bind to germinant receptors in spores’ inner membrane and this interaction triggers the release of dipicolinic acid and cations from the spore core and their replacement by water. *Bacillus subtilis* spores contain three functional germinant receptors encoded by the *gerA*, *gerB*, and *gerK* operons. The GerA germinant receptor alone triggers germination with L-valine or L-alanine, and the GerB and GerK germinant receptors together trigger germination with a mixture of L-asparagine, D-glucose, D-fructose and KCl (AGFK). Recently, it was reported that the *B. subtilis gerW* gene is expressed only during sporulation in developing spores, and that GerW is essential for L-alanine germination of *B. subtilis* spores but not for germination with AGFK. However, we now find that loss of the *B. subtilis gerW* gene had no significant effects on: i) rates of spore germination with L-alanine; ii) spores’ levels of germination proteins including GerA germinant receptor subunits; iii) AGFK germination; iv) spore germination by germinant receptor-independent pathways; and v) outgrowth of germinated spores. Studies in *Bacillus megaterium* did find that *gerW* was expressed in the developing spore during sporulation, and in a temperature-dependent manner. However, disruption of *gerW* again had no effect on the germination of *B. megaterium* spores, whether germination was triggered via germinant receptor-dependent or germinant receptor-independent pathways.

## Introduction

*Bacillus* species have two alternative life cycles. In the vegetative cycle with abundant nutrients, these organisms replicate their chromosome and divide by binary fission into two equivalent daughter cells [1]. However, in response to nutrient limitation, a morphologically distinct cell type called a spore is formed through a process termed sporulation [2]. Spores of *Bacillus* and *Clostridium* species are metabolically dormant with extreme resistance to environmental stresses, and are capable of surviving extreme temperatures, desiccation, chemical agents, and UV- and γ-radiation [3].

Spores are dormant and by themselves cannot cause deleterious effects. However, spores sense their environment and when specific signaling molecules, most often specific nutrients are again present, spores can return to life rapidly through germination. An early event in germination is the release from the spore core of large amounts of the 1:1 chelate of Ca^2+^ and dipicolinic acid (CaDPA) through inner membrane (IM) channels composed at least in part of SpoVA proteins. This is followed by hydrolysis of spore cortex peptidoglycan and expansion of the spore core. Finally metabolism and macromolecular synthesis convert the dormant spore into a growing cell in outgrowth [4]. Nutrients generally trigger spore germination through interactions with proteins called germinant receptors (GRs) located in spores’ IM. *Bacillus* spores most often have multiple GRs, each with a different specificity for a nutrient germinant or nutrient germinant mixture. GRs are generally encoded by tricistronic operons encoding GRs’ A-, B-, and C-subunits. The A and B subunits are likely integral IM proteins and the C subunit is a lipid-anchored peripheral IM protein [5,6]. By far the best-studied *Bacillus* species is *Bacillus subtilis*, and this species’ genome contains five tricistronic operons encoding GRs. The GerA GR responds to L-alanine or L-valine alone, while the GerB and GerK GRs are both required for germination with a mixture of L-asparagine plus D-glucose, D-fructose, and K^+^ ions (termed AGFK) [6,7]. The GRs encoded by the other two operons have no known function.

Another protein involved in triggering of spore germination is GerD, a peripheral IM lipoprotein. GerD colocalizes with GRs in a single cluster in dormant spores. These clusters represent a functional germination unit or “germinosome”, facilitating spores’ rapid and cooperative response to nutrients [8]. Recently, the GerW protein made in the developing spore was reported to be important in triggering of *B. subtilis* spore germination with L-alanine, as rates of L-alanine germination of GerW-deficient spores were > 10-fold lower than those of wild-type spores [9]. In contrast, rates of AGFK germination of *gerW*-deficient spores were almost identical to those of wild-type spores [9]. In the current work, we have examined the effects of the GerW protein on rates of germination in spores of two *Bacillus* species and levels of germination proteins in *B. subtilis* spores. Surprisingly, the absence of the GerW protein had no significant effects on *B. subtilis* spore germination rates with either L-alanine or AGFK or the levels of germination proteins. *B. megaterium* QM B1551 *gerW*-deficient spores also germinated efficiently in response to nutrient and non-nutrient stimulants. Collectively, these results indicate that GerW has no role in the germination of spores of the two species examined in this work, and in all likelihood *Bacillus* spores in general.

## Materials and Methods

*B. subtilis* strains used in this work are isogenic derivatives of strain PS832 (wild-type), a prototrophic laboratory derivative of strain 168 (Table 1). To obtain the *gerW B. subtilis* strain PS4389 most of the *gerW* coding sequence was replaced by a chloramphenicol resistance (Cm^r^) cassette as follows. The region between bp-124 to +115 relative to the *gerW* translation start (+1) was PCR amplified from *B. subtilis* PS832 DNA using primers containing *BamH*I and *Pst*I sites (Start Forward and Start Reverse primers; all primer sequences are available upon request). The purified PCR product was digested with *BamH*I and *Pst*I and ligated to a similarly digested modified pBluescript II KS plasmid that has a Cm^r^ cassette between *EcoR*I and *EcoR*V sites. The ligation reaction was used to transform *Escherichia coli* DH5α to ampicillin resistance (Amp^r^) giving plasmid pJCM1. The presence of the appropriate *gerW* fragment in pJCM1, as well as in all other plasmid constructs was confirmed by PCR and restriction enzyme digestion. The region between bp +290 to +891 relative to the *gerW* translation start codon of the *gerW* gene coding and downstream region was PCR amplified from *B. subtilis* PS832 DNA using primers containing *Hind*III and *Kpn*I sites (EndP Forward and EndP Reverse primers). The purified PCR product was digested with *Hind*III and *Kpn*I, and ligated to *Hind*III and *Kpn*I digested plasmid pJCM1. This ligation reaction transformed *E. coli* DH5α to Amp^r^ giving plasmid pJCM2. Plasmid pJCM2 transformed *B. subtilis* strain PS832 to Cm^r^ giving strain PS4389 (*gerW*1). The expected genome structure in the *gerW* region of strain PS4389, as well as in the other *B. subtilis gerW* mutant strain described below, was confirmed by PCR and DNA sequencing (data not shown). *B. subtilis* strain PS4399 encoding only the first 6 aa of the GerW protein was constructed as follows. The region between bp-124 to +18 relative to the *gerW* translation start codon was PCR amplified from *B. subtilis* PS832 DNA using primers containing *BamH*I and *Pst*I sites (Start Forward and Start 2 Reverse primers). The purified PCR product was digested with *BamH*I and *Pst*I and ligated to similarly digested plasmid pJCM2 from which bp-124 to + 115 of *gerW* had been removed. The ligation reaction transformed *E. coli* DH5α to Amp^r^ giving plasmid pJCM3. This plasmid transformed *B. subtilis* strain PS832 to Cm^r^ giving strain PS4399 (*gerW*2).

**Table 1:** Bacterial strains and plasmids used in this study.

Strains	Relevant genotype, phenotype or description^a^	Reference or source
***Bacillus subtilis***		
PS832	Wild-type prototroph	Lab strain
PS4389	*gerW*1 (encodes 38 aa of GerW) Cm^r^	This work
PS4399	*gerW*2 (encodes 6 aa of GerW) Cm^r^	This work
***Bacillus megaterium***		
QM B1551	Wild-type	Pat Vary
GC618	*gerU*::pNFD13 Km^r^	10
GC900	*gerW* pHT-GerU* Zn^r^ Er^r^	This work
GC918	*gerW*::pNFD13 Km^r^	This work
GC919	*gerD*::pNFD13 Km^r^	This work
***Escherichia coli***		
DH5α	Competent cells	Lab strain
**Plasmids**		
Modified pBluescript II KS	Amp^r^ Cm^r^	Lab plasmid
pJCM 1	*gerW* (-124 to +115) Amp^r^ Cm^r^	This work
pJCM 2	Plasmid with Δ*gerW*1 Amp^r^ Cm^r^-	This work
pJCM 3	Plasmid with Δ*gerW*2 Amp^r^ Cm^r^	This work
pGEM-3Z	Amp^r^	Promega
P7Z6	Amp^r^ Zn^r^	BGSC^b^
pUCTV2	Amp^r^ Tc^r^ ts replication	11
pDONRtet	Gateway entry plasmid Tc^r^	12
pNFD13	Vector to create *lacZ* fusions Km^r^	12
pUC-Δ*gerW*::Zn	*B. megaterium* Δ*gerW* Amp^r^ Tc^r^ Zn^r^	This work
pHT-GerU*	encodes GerU* GR genes Er^r^	13

^a^Abbreviations used are: ts, temperature sensitive; Amp^r^, ampicillin resistance (100 μg/ml); Cm^r^, chloramphenicol resistance (5 μg/ml); Er^r^, erythromycin resistance (1 μg/ml); Km^r^, kanamycin resistance (10 μg/ml); Tc^r^, tetracycline resistance (12.5 μg/ml); Zn^r^, zeocin resistance (20 μg/ml).

^b^Bacillus Genetic Stock Center

The *B. megaterium gerW* strain (GC900) was constructed using PCR to initially amplify a 1075 bp DNA fragment encompassing the *gerW* ORF (BMQ_4796), using primers with 5′-*EcoR*I restriction sites. The purified *EcoR*I digested PCR product was ligated with *EcoR*I linearised pGEM-3Z, giving plasmid pGEM-*gerW* in *E. coli*. An inverse PCR using pGEM-*gerW* as template and with appropriate primers incorporated a 60 bp deletion towards the middle of the *gerW* ORF and with 5′-*Nco*I sites. The purified inverse PCR product was ligated with a zeocin resistance (Zn^r^) cassette (excised from plasmid p7Z6). Plasmid pGEM-Δ*gerW*::Zn was isolated from *E. coli*, and used as a template for a PCR reaction amplifying the Δ*gerW*::Zn cassette, using primers adding 5′-*Mfe*I sites. This cassette was digested with *Mfe*1 and ligated with *EcoR*I digested pUCTV2 [10], giving plasmid pUC-Δ*gerW*::Zn. This plasmid was used to transform *B. megaterium* QM B1551 protoplasts to tetracycline resistance (Tc^r^), using the polyethyleneglycol (PEG)-mediated procedure described previously [14]. A colony that had replaced the native *gerW* locus with the Δ*gerW*::Zn cassette via allelic exchange, conferring a Tc^s^ Zn^r^ phenotype, was isolated after repeated sub-culture of single-crossover cells at 42°C in the absence of antibiotic. PCR was used to verify the correct construction of the mutant strain. These analyses revealed that the native pBM700 plasmid, which carries the GerU GR structural genes, had been excised during mutant construction procedures. To circumvent this loss, plasmid pHT-GerU*, which is stable in *B. megaterium* at low copy number, and which encodes functional *gerU* GR genes plus regulatory sequences [13], was introduced by PEG-mediated transformation into the *B. megaterium gerW* strain.

A *B. megaterium* strain bearing a transcriptional fusion between the *gerW* ORF and *E. coli lacZ* was constructed essentially as described previously, using the Gateway cloning technique [10,12]. The *gerW* ORF was amplified by PCR using primers designed to introduce 5′ attB sites, and then purified and cloned into pDONRtet using the Gateway BP reaction mix (Life Technologies, Paisley, UK). Purified intermediate plasmid DNA was isolated from transformed *E. coli* and then employed in a Gateway LR reaction to create a pNFD13 derived *gerW*-*lacZ* plasmid. *B. megaterium* QM B1551 was transformed to kanamycin resistance (Km^r^) with this plasmid using the PEG-mediated transformation method. A colony that had undergone homologous recombination to create a *gerW-lacZ* fusion strain (GC918) was isolated after incubation at 42°C, and its correct construction was verified by PCR. Lysates of spores (10^9^) of strain GC918 were used in subsequent β-galactosidase assays as described previously [10] along with the same amounts of spores of *B. megaterium* strains carrying *gerU-lacZ* (strain GC618) or *gerD-lacZ* (strain GC919) for comparative purposes. The *B. megaterium gerD*-*lacZ* strain (*gerD* is encoded by BMQ_0176) was created in a similar manner to the *gerW*-*lacZ* strain.

Spores of *B. subtilis* strains were routinely prepared at 37°C on 2x Schaeffer’s-glucose plates without antibiotics as described previously [15,16]. After incubation for ~ 5 d, the spores were scraped from plates, and washed with water by repeated centrifugation with intermittent sonication treatment. In one experiment, *B. subtilis* spores were prepared in liquid Schaeffer Sporulation medium as described previously [17], and spores were purified as described above. *B. megaterium* spores were prepared at 30°C in supplemented nutrient broth (SNB), and purified by repeated rounds of centrifugation and washing with ice-cold deionized water as described previously [11]. All spore preparations used in this work were free (~ 95%) from growing or sporulating cells and germinated spores as determined by phase-contrast microscopy.

*B. subtilis* spores were germinated following heat activation (30 min; 75°C) and cooling on ice for 10 min. Spores at an optical density at 600 nm (OD_600_) of 0.5 were germinated for 2.5 h at 37°C in 200 μl of 25 mM K-Hepes buffer (pH 7.4) with various concentrations of L-alanine or 10 mM L-valine, or with 10 mM of each AGFK component. All germination experiments were carried out in duplicate. Spore germination was routinely monitored by measuring the release of the spores’ large depot of DPA by inclusion of 50 μM TbCl_3_ in germination mixtures and measuring Tb-DPA fluorometrically in a multiwell plate reader as described previously [18]. Germination of spores with a coat defect due to chemical decoating is very strongly inhibited by TbCl_3_ [19]. Consequently, decoated spores prepared as described previously [19] were germinated as described above, but without Tb^3+^ present from the initiation of germination. Instead, at various times after germination was initiated, aliquots of the germinating culture were centrifuged in a microcentrifuge, the supernatant fluid made 50 μM in TbCl_3_ and Tb-DPA fluorescence was measured as described previously [19,20]. Spore germination was also routinely monitored at the end of germination incubations by phase-contrast microscopy. The total amount of DPA present in spores was assessed by Tb-DPA fluorescence after DPA had been released from spores by boiling as described previously [18,19]. In some experiments, spore germination was also monitored by the fall in optical density of spore cultures as described previously [9]. All *B. subtilis* GR-dependent spore germination experiments were carried out on multiple independent spore preparations prepared in different laboratories with essentially identical results.

*B. subtilis* spores that were not heat-activated were also germinated with GR-independent germinants as follows: i) at 50°C in 25 mM K-Hepes buffer (pH 7.4) and 1 mM dodecylamine, with spores at an OD_600_ of 2; and ii) at 23°C in 60 mM CaDPA made to pH 7.5 with Tris base, with spores at an OD_600_ of 2 [5]. Germination of spores with CaDPA and dodecylamine was monitored by examining ~ 100 individual spores at various times by phase-contrast microscopy [7].

Outgrowth of heat-activated *B. subtilis* spores was carried out at 37°C in 2x yeast tryptone (2x YT) medium containing 5 mM L-alanine and (per L) 16 g tryptone, 10 g yeast extract, 5 g NaCl. Spores were added to an OD_600_ of 0.5 and the OD_600_ of cultures were followed over time [21]. Finally, to determine spore viability, both PS832 and PS4389 spores at an OD_600_ of 1.0 were heat activated, cooled, spores spotted on LB medium plates with the appropriate antibiotic, plates incubated for 24 h at 37°C and colonies were counted [21].

*B. megaterium* spore germination was followed by monitoring the absorbance at 600 nm of heat-shocked (60°C, 10 min) spores suspended at an OD_600_ of ~ 0.4 in 5 mM Tris-HCl, pH 7.8, plus 0.1–25 mM glucose or proline. Germination assays were conducted in triplicate, in 96-well plates incubated at 30°C in a PerkinElmer EnVision-Xcite multilabel plate reader fitted with a 600 nm photometric filter. Similar absorbance-based assays were conducted with non-heat shocked spores incubated in either 60 mM CaDPA at 30°C or 1 mM dodecylamine at 40°C. All experiments were conducted with at least two independently prepared batches of spores with essentially identical results. Spore viability was assessed by plating serial dilutions of heat-shocked spores on solid LB medium plates which were incubated at 30°C overnight before determining the percent viability of *gerW* spores compared to wild-type spores in which 1 OD_600_ unit is equal to ~ 10^8^ CFU ml^-1^.

Levels of GR, GerD and SpoVAD proteins, which are present largely or completely in spores’ IM [22–26], were measured in lysates of spores by western blot analysis using polyclonal rabbit antisera against the various proteins and a secondary antiserum as described previously [7,21,27,28]. In brief, spores were decoated, ruptured by lysozyme digestion, and sonicated briefly with glass beads present to obtain spore lysates. Aliquots of the lysates were then run on SDS-polyacrylamide electrophoresis (SDS-PAGE) and the gels were stained with Coomassie blue to determine how much of the lysates needed to be run to load equal amounts of protein. Equal amounts of the lysates were run on SDS-PAGE, proteins transferred to a polyvinylidenedifluoride (PVDF) membrane, and antigens on the membrane were detected as described previously [10,21,27,28]. Following development of these western blots, the membrane was stripped and then reprobed with another antiserum as described previously [21,28].

## Results

**Fig 1:**
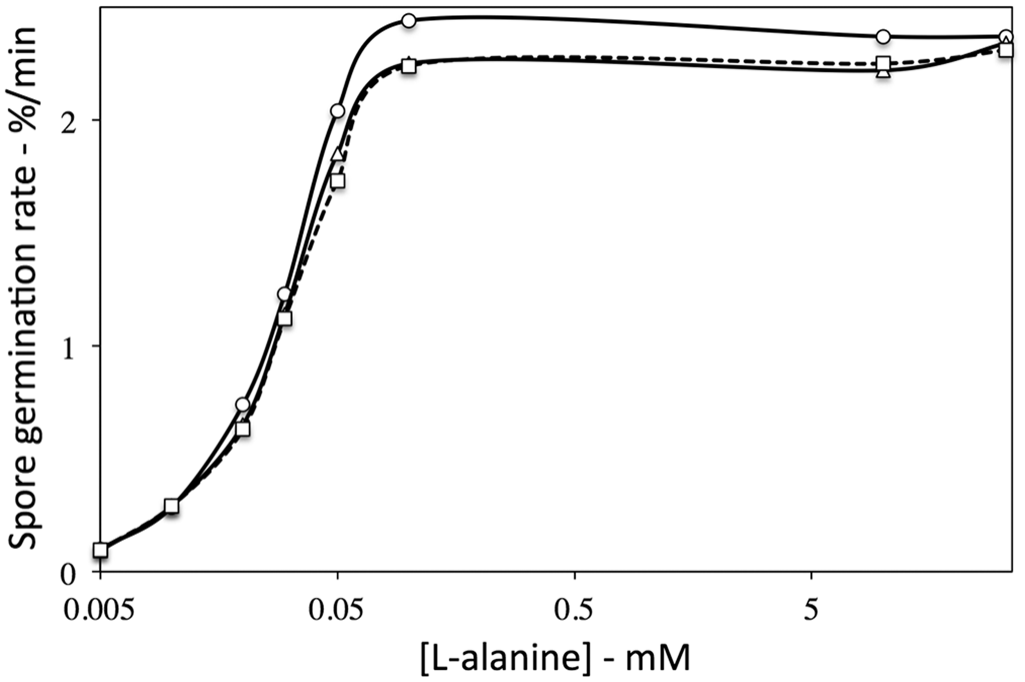
Rates of germination of wild-type and *gerW B*. *subtilis* spores with various L-alanine concentrations. Heat shocked wild-type (PS832, ◯), *gerW*1 (PS4389, □) and *gerW*2 (PS4399, Δ) *B. subtilis* spores were germinated with various L-alanine concentrations and DPA released was monitored as described in Methods. Rates of spore germination are averages of values determined from maximum slopes of DPA release curves in two separate experiments, and these values varied by < 15%.

It was reported recently that the rate of L-alanine germination of *gerW B. subtilis* spores was > 10-fold slower than that of the wild-type spores, although AGFK germination of *gerW* spores was normal [9]. To further investigate the effect of a *gerW* mutation on spore germination, we replaced the great majority of the *gerW* coding sequence in *B. subtilis* strain PS832 (wild-type) by a Cm^r^ cassette giving *B. subtilis* strains PS4389 (*gerW*1) (retains 38 N-terminal GerW aa) and PS4399 (*gerW*2) (retains 6 N-terminal GerW aa). Strain PS4389 was generated first, and when spores of this strain were found to germinate normally with L-alanine, we also prepared strain PS4399 to eliminate the possibility that the N-terminal 38 aa of GerW were sufficient for its function. Multiple germination experiments with wild-type and PS4389 and PS4399 spores indicated that rates of *gerW* mutant spores’ germination with AGFK were essentially identical to those of wild-type spores (Table 2), as reported previously [9]. Surprisingly the PS4389 and PS4399 spores also germinated like wild-type spores with either L-alanine or L-valine via the GerA GR (Table 2). Based on these experiments, there were no statistically significant differences between rates of wild-type and *gerW* spore germination with saturating levels of different nutrient germinants. In addition, wild-type and *gerW* spores prepared in liquid Schaeffer’s-glucose sporulation medium or in liquid Spizizen’s minimal medium [28,29] also germinated essentially identically with L-alanine (data not shown). Chemically decoated wild-type and PS4389 spores prepared as described previously [19] also germinated identically with L-alanine (data not shown), and the intact *gerW* spores’ germination with different L-alanine concentrations was also essentially identical to that of wild-type spores (Fig. 1).

**Table 2:** Rates of germination of wild-type and *gerW B. subtilis* spores with L-alanine, L-valine or AGFK.

Germinants:	10 mM L-alanine	10 mM L-valine	10 mM AGFK
Spores		Spore germinationrate—%/min	
PS832 (wild-type)	2.6	2.5	0.75
PS4389 (*gerW*1)	2.4	2.3	0.8
PS4399 (gerW2)	2.5	2.4	0.78

Spores of various strains were germinated with saturating levels of different germinants, and spore germination was monitored by DPA release as described in Methods. Rates of spore germination were determined from plots of DPA release as a function of time. Values shown are averages of values in two independent experiments with the same spore preparations that differed by < 12%.

In addition to nutrient germinants that trigger spore germination via GRs, we also measured the germination of wild-type and *gerW B. subtilis* spores with CaDPA and dodecylamine, two agents that trigger spore germination without GR involvement. Again, we observed no significant difference between *gerW* and wild-type spores in their germination with either CaDPA or dodecylamine (data not shown).

**Fig 2:**
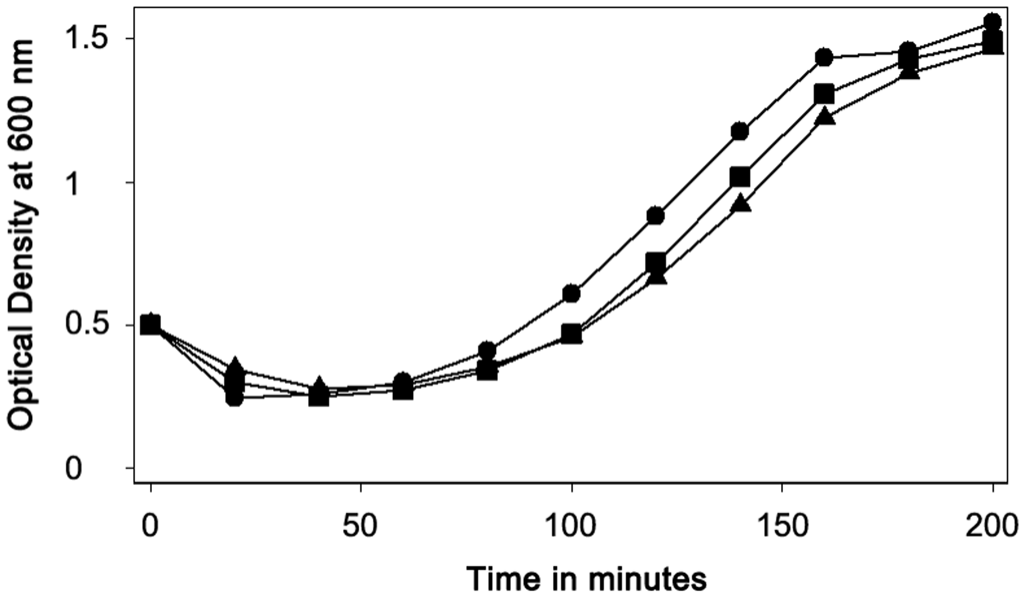
Germination and outgrowth of wild-type and *gerW B*. *subtilis* spores. Spores of *B. subtilis* strains PS832 (wild-type; ●), PS4389 (*gerW*1; ■) and PS4399 (*gerW*2, ▲) were heat shocked, cooled, and incubated with shaking at 37°C and an initial OD_600_ of 0.5 in 2xYT medium plus 5 mM L-alanine, and the OD_600_ of the cultures was measured.

We also measured the ability of wild-type and *gerW* spores to return to active growth after spore germination in a complete nutrient medium with L-alanine added to ensure rapid spore germination, and observed that both types of spores had similar rates of outgrowth (Fig. 2). In addition, the viability of wild-type and *gerW* spores was essentially identical when heat activated spores were spotted on LB medium plates (data not shown).

**Fig 3:**
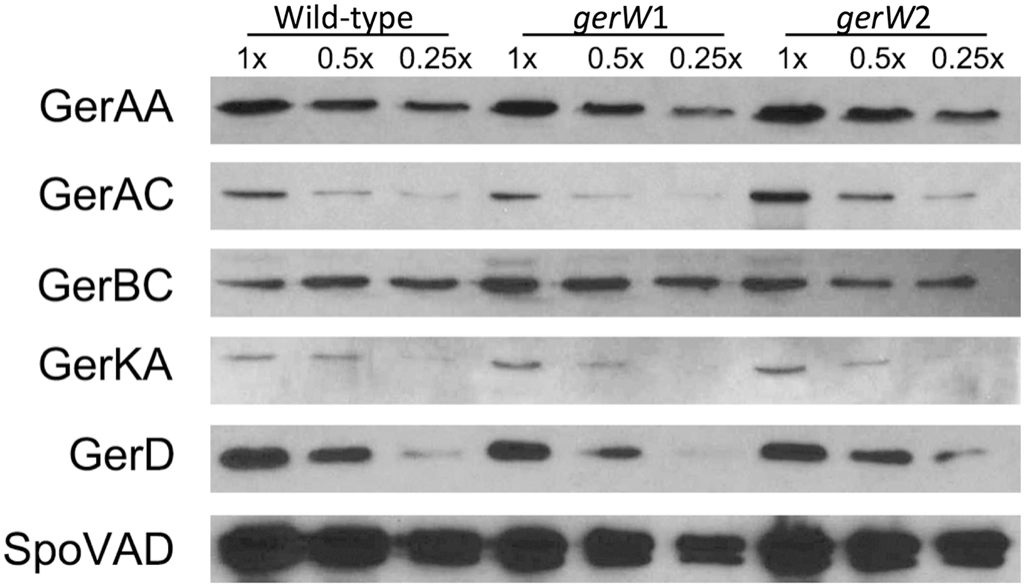
Levels of GR subunits, GerD and SpoVAD in wild-type and *gerW B*. *subtilis* spores. Aliquots of equal amounts of protein in lysates from spores of *B. subtilis* strains PS832 (wild-type), PS4389 (*gerW*1) or PS4399 (*gerW*2) were run on SDS-PAGE, proteins transferred to PVDF paper, and these western blots were probed with antisera against various proteins as described in Methods. The amount of protein in lysates in the 1x samples from wild-type and *gerW* spores was identical.

The levels of GR subunits, GerD and SpoVAD proteins were also determined in lysates of spores by western blot analysis. Some modest differences were observed between levels of these proteins in PS832 (wild-type), PS4389 (*gerW*1) and PS4399 (*gerW*2) spores in some experiments, although these were generally ≤ 2-fold (Fig. 3). In addition, when blots from multiple replicate experiments were compared, there were on average ≤ 25% differences in the intensities of different germination proteins from wild-type and *gerW* spores (data not shown). In general, the similar levels of GR proteins and GerD in wild-type and *gerW* spores was consistent with the similar rates of germination of wild-type and *gerW* spores with all GR-dependent germinants (Table 2).

Bioinformatic analyses revealed that the *B. megaterium* QM B1551 genome also contains a single orthologue of *B. subtilis gerW*, encoded at locus BMQ_4796. The predicted protein shares 70% amino acid identity with its *B. subtilis* counterpart, with most variance occurring in an ~20 aa sequence towards the central region of the protein (data not shown). Analysis of lysates from disrupted purified *B. megaterium gerW-lacZ* spores revealed β-galactosidase activity, levels of which were dependent upon the temperature of sporulation (Table 3). Spores bearing a *lacZ* fusion to the GR gene, *gerUA*, also showed temperature dependent levels of expression as reported previously [10]. Collectively, the β-galactosidase assays indicate that GerD is expressed at a higher level than GerW or GerU at 22°C, while GerW and GerD are expressed at similar levels at 30 and 37°C.

**Table 3:** β-Galactosidase activity from *gerW-*, *gerD- and gerUA-lacZ* transcriptional fusions in *B. megaterium* spores prepared at different temperatures.

Strain	Genotype	β-Galactosidase activity (relative fluorescence units)^1^		
		Sporulation temperature ^1^		
		22°C	30°C	37°C
GC918	*gerW-lacZ*	9.5E+03	3.3E+04	2.9E+04
GC919	*gerD-lacZ*	3.6E+04	3.6E+04	3.0E+04
GC618	*gerU-lacZ*	4.2E+03	3.2E+03	7.9E+02

Spores were prepared at three different temperatures for each strain as described in Methods, and the β-galactosidase activity in lysates from 10^9^ spores of each strain was measured in triplicate. Similar values were obtained with at least one other independently prepared batch of spores. Values have been corrected for wild-type spore background levels of fluorescence which were always < 500 relative fluorescence units. Standard deviations for all values were < 15%.

Overall, in *B. megaterium*: i) the expression of GerW-LacZ in spores; ii) the identification of a putative σ^F^ promoter sequence upstream of BMQ_4796 with sequence homology to the *B. subtilis gerW* promoter region and approximately the same spacing between the two promoters and the translation start sites [9] (data not shown); and iii) the lack of detection of *gerW* mRNA in vegetative cells by RT-PCR (data not shown) are all consistent with forespore-specific expression of *gerW*, as observed previously in *B. subtilis* [9]. However, there were differences in the expression of the *gerW*, g*erD* and *gerUA* genes as a function of sporulation temperature (see Discussion).

**Fig 4:**
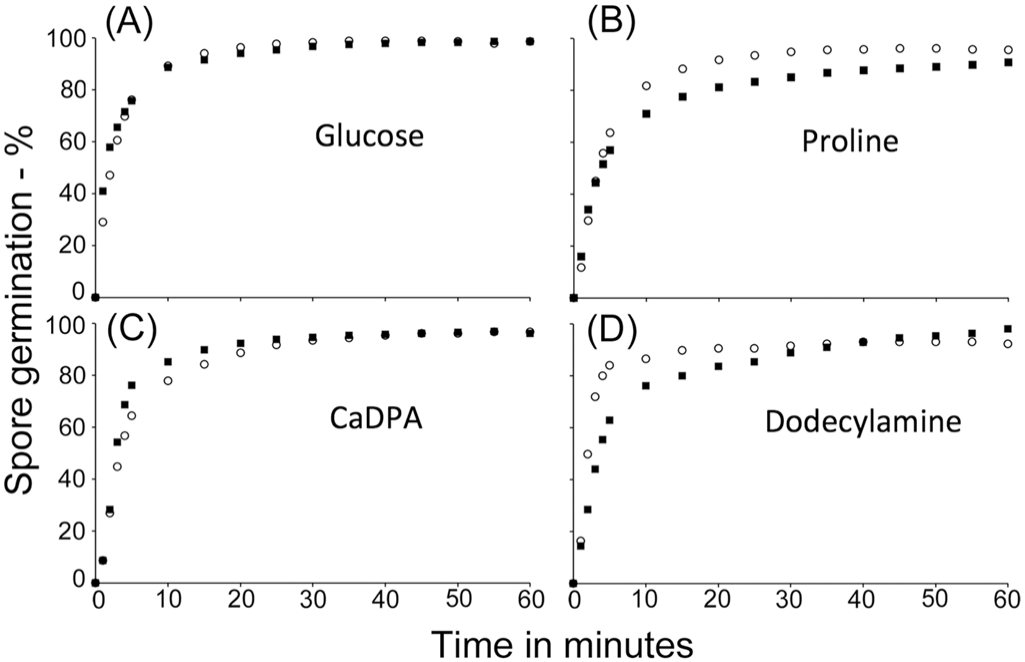
Germination of wild-type and *gerW B*. *megaterium* spores with various germinants. *B. megaterium* spores were germinated in 5 mM Tris-HCl, pH 7.8 plus (A) 10 mM glucose, (B) 10 mM proline, (C) 60 mM CaDPA, or (D) 1 mM dodecylamine. Spores were heat shocked at 60°C for 10 min prior to incubation in glucose or proline-supplemented germination buffer, but not for CaDPA or dodecylamine germination, and the progress of spore germination was monitored as described in Methods. Symbols used are: (◯), wild type spores; and (■), *gerW* spores.

In order to also investigate the role, if any, of GerW in the germination of *B. megaterium* spores, the *gerW* gene was disrupted with a Zn cassette by allelic exchange, which introduced a short deletion in the *gerW* ORF. The resultant strain was found to have excised plasmid pBM700 during strain construction, hence plasmid pHT-GerU* was introduced by PEG-mediated transformation to restore the *gerU* GR genes and their regulatory sequences. The *B. megaterium gerW* pHT-GerU* strain (GC900) sporulated normally (data not shown) and the spores were then examined for germination efficiency in response to nutrient and non-nutrient stimuli. *B. megaterium gerW* spores were observed to germinate essentially with an identical efficiency to wild-type spores in response to either glucose or proline, including at sub-optimal germinant concentrations (Fig. 4A,B; Table 4). Similarly, spores with disrupted *gerW* displayed essentially wild-type germination with the GR-independent germinants CaDPA and dodecylamine (Fig. 4C,D). Additionally, *B. megaterium* GerK^+^ spores, in which only the *gerK* GR operon is intact, germinated normally when plated on solid LB medium whether *gerW* was disrupted or not, as did the wild-type spores containing only the *gerK* GR operon (data not shown).

**Table 4:** Maximum rates of germination of wild-type and *gerW B. megaterium* spores in response to varying concentrations of glucose or proline.

Germinant:	Glucose mM				Proline mM			
	**0.1**	**1**	**10**	**25**	**0.1**	**1**	**10**	**25**
**Strain**	**Maximum rate of germination—% OD_600_/min**							
QM B1551 (wt)	0.67	3.5	15.3	14.1	0.68	3.5	10.4	10.7
GC900 (*gerW*)	0.59	3.5	14.6	11.8	0.50	2.3	11.1	11.2

*B. megaterium* spores were heat shocked and incubated in 5 mM Tris-HCl, pH 7.8 plus the designated concentration of glucose or proline, and germination was monitored by measuring OD_600_ loss as described in Methods. The maximum % OD_600_ loss per min was calculated from plots of OD_600_ versus time. Values shown are the averages of results from three independent experiments conducted with the same spore preparations, and the standard deviations were <15% of the averages.

## Discussion

Clearly, the major conclusion from the current work is that GerW is not essential for the germination of spores of two *Bacillus* species, either *B. subtilis* spores with L-alanine or AGFK, and *B. megaterium* spores with either GR-dependent or GR-independent germinants. The obvious question based on the new findings is why GerW was previously found to be essential for normal *B. subtilis* spore germination with L-alanine via the GerA GR [9]. The answer to this question is not completely clear. However, it seems most likely that the original transformation to generate a *gerW* deletion mutation used a laboratory *B. subtilis* 168 strain [30], while the wild-type strain was strain 1A1 from the *Bacillus* Genetic Stock Center (BGSC). Unfortunately, the *B. subtilis* laboratory 168 strain used in the initial communication now appears to have had one or more mutations that significantly reduce its spores’ germination with L-alanine even without deletion of *gerW*. In contrast, spores of the PS832 168 strain germinate very rapidly with L-alanine.

In addition to the major conclusion discussed above, there are several other notable points pertinent to the current work as follows. 1) Expression of *gerW* in *B. megaterium* and *B. subtilis* is forespore-specific during sporulation, as is that of many genes involved in spore-specific properties. However, in *B. megaterium*, *gerW* expression displayed a rather different response to sporulation temperature than did two other forespore-specific genes, *gerD* and *gerUA*. Differences in the regulation of these three genes as a function of temperature may reflect differences in the RNA polymerase σ factors that recognize these genes, σ^F^ for *gerW* and σ^G^ for *gerD* and *gerUA*, as well as likely additional regulators of σ^G^-dependent genes such as SpoVT [14,31,32]. 2) While GerW does not play an obvious role in *Bacillus* spore germination, at least in *B. megaterium* and *B. subtilis*, an important question is what does this protein do. The GerW amino acid sequence suggests the protein is soluble, and GerW is present in the soluble fraction of disrupted *B. subtilis* spores [9]. In addition, a Blast search of the NCBI microbial genomes database readily detects obvious GerW homologs in the spore forming members of the order *Bacillales*, but also in the order *Clostridiales*. The latter information, as well as that *gerW* is expressed only in the developing spore suggests that GerW plays some important role in dormant spore properties. However, this role remains to be determined.
